# Delayed Evolution of Neurofibromatosis Type 1 in a Child With an Isolated Cervical Plexiform Neurofibroma Initially Mimicking Cystic Lymphangioma

**DOI:** 10.7759/cureus.112316

**Published:** 2026-07-09

**Authors:** Deeva Patel, Saurabh H Gandhi, Rinkal Patel

**Affiliations:** 1 Medicine and Surgery, B. J. Medical College, Ahmedabad, IND; 2 Otolaryngology, Smt. N. H. L. Municipal Medical College, Ahmedabad, IND; 3 Pathology, B. J. Medical College, Ahmedabad, IND

**Keywords:** café-au-lait spots, cystic lymphangioma, lisch nodule, neurofibromatosis 1, plexiform neurofibroma, s100 protein

## Abstract

We report the case of an 11-year-old female who presented with a progressively enlarging cystic neck swelling. CT demonstrated a multiloculated cystic lesion involving the right cervical soft tissue planes, suggestive of a cystic lymphangioma. There was no significant family history or clinical evidence of neurofibromatosis type 1 (NF1) at presentation. Surgical excision was performed, and histopathological examination with S100 immunohistochemistry confirmed the diagnosis of plexiform neurofibroma. At the one-year follow-up, the patient developed multiple café-au-lait macules and Lisch nodules of the iris, fulfilling the diagnostic criteria for NF1. The delayed appearance of these characteristic features established the diagnosis of evolving sporadic NF1. This case highlights that an isolated plexiform neurofibroma may be the initial manifestation of NF1 and can clinically and radiologically mimic other cervical soft tissue lesions. Careful long-term clinical, dermatological, and ophthalmological follow-up is essential for the timely recognition of additional diagnostic features and appropriate surveillance.

## Introduction

Neurofibromatosis type 1 (NF1), also known as von Recklinghausen disease, is an autosomal dominant neurocutaneous disorder with an incidence of approximately one in 2,500-3,000 live births. NF1 is caused by pathogenic variants in the NF1 tumor suppressor gene located on chromosome 17q11.2, resulting in loss of neurofibromin function and dysregulation of the RAS/MAPK signaling pathway [1]. Nearly 50% of cases arise from de novo mutations without a positive family history [2].

NF1 is characterized by multisystem involvement, including cutaneous, neurological, skeletal, and ophthalmological manifestations. Diagnostic criteria established by the National Institutes of Health include the following: six or more café-au-lait macules >5 mm in greatest diameter in prepubertal individuals or >15 mm in greatest diameter in postpubertal individuals; two or more neurofibromas of any type or one plexiform neurofibroma; freckling in the axillary or inguinal region; optic glioma; two or more Lisch nodules; distinctive osseous lesions, such as sphenoid dysplasia or thinning of the long bone cortex with or without pseudoarthrosis; or a similar history in a first-degree relative (parent, sibling, or offspring) [2]. Among these, plexiform neurofibroma is regarded as one of the most characteristic lesions of NF1 and occurs in nearly 30% of affected patients [3].

Macroscopically, plexiform neurofibromas are large lesions that involve long segments of a nerve, distorting it into a characteristic “bag of worms” appearance [4]. Microscopically, plexiform neurofibromas are benign peripheral nerve sheath tumors composed of tortuous masses of expanded nerve branches with associated proliferation of Schwann cells, fibroblasts, and perineural cells. They frequently demonstrate immunoreactivity for S100 protein [1]. Although commonly associated with NF1, isolated plexiform neurofibromas without other clinical manifestations have also been reported and may pose significant diagnostic difficulty.

Clinically and radiologically, plexiform neurofibromas may mimic vascular malformations, hemangiomas, lymphangiomas, or other soft tissue tumors, particularly in children and young adults [5]. The absence of classic cutaneous findings or a family history at presentation can delay recognition of NF1 until additional manifestations appear during follow-up. Because NF1 demonstrates age-dependent penetrance and variable expressivity, some patients initially present with only a solitary plexiform neurofibroma before developing other diagnostic features [2].

We report a rare case of a cervical plexiform neurofibroma initially presenting without definitive features of NF1 and clinically mimicking a lymphangioma. During follow-up, the patient subsequently developed characteristic skin lesions and iris hamartomas (Lisch nodules), ultimately fulfilling the diagnostic criteria for NF1. This case highlights the evolving clinical spectrum of NF1 and emphasizes the importance of careful long-term surveillance in patients presenting with an isolated plexiform neurofibroma.

## Case presentation

An 11-year-old female presented with a progressively enlarging swelling in the cervical region for five years. The swelling was located on the right side of the neck and was soft to firm, extending from below the angle of the mandible to the posterior border of the sternocleidomastoid muscle. It was multiloculated and measured approximately 40 × 24 × 85 mm. There was no history of a similar illness in family members. No definite café-au-lait macules, axillary freckling, cutaneous neurofibromas, skeletal deformities, or ophthalmological abnormalities were documented at the initial presentation.

Radiological evaluation with CT revealed a multiloculated cystic lesion involving the right cervical soft tissue planes and the right sternocleidomastoid muscle, suggestive of a cystic lymphangioma.

**Figure 1:**
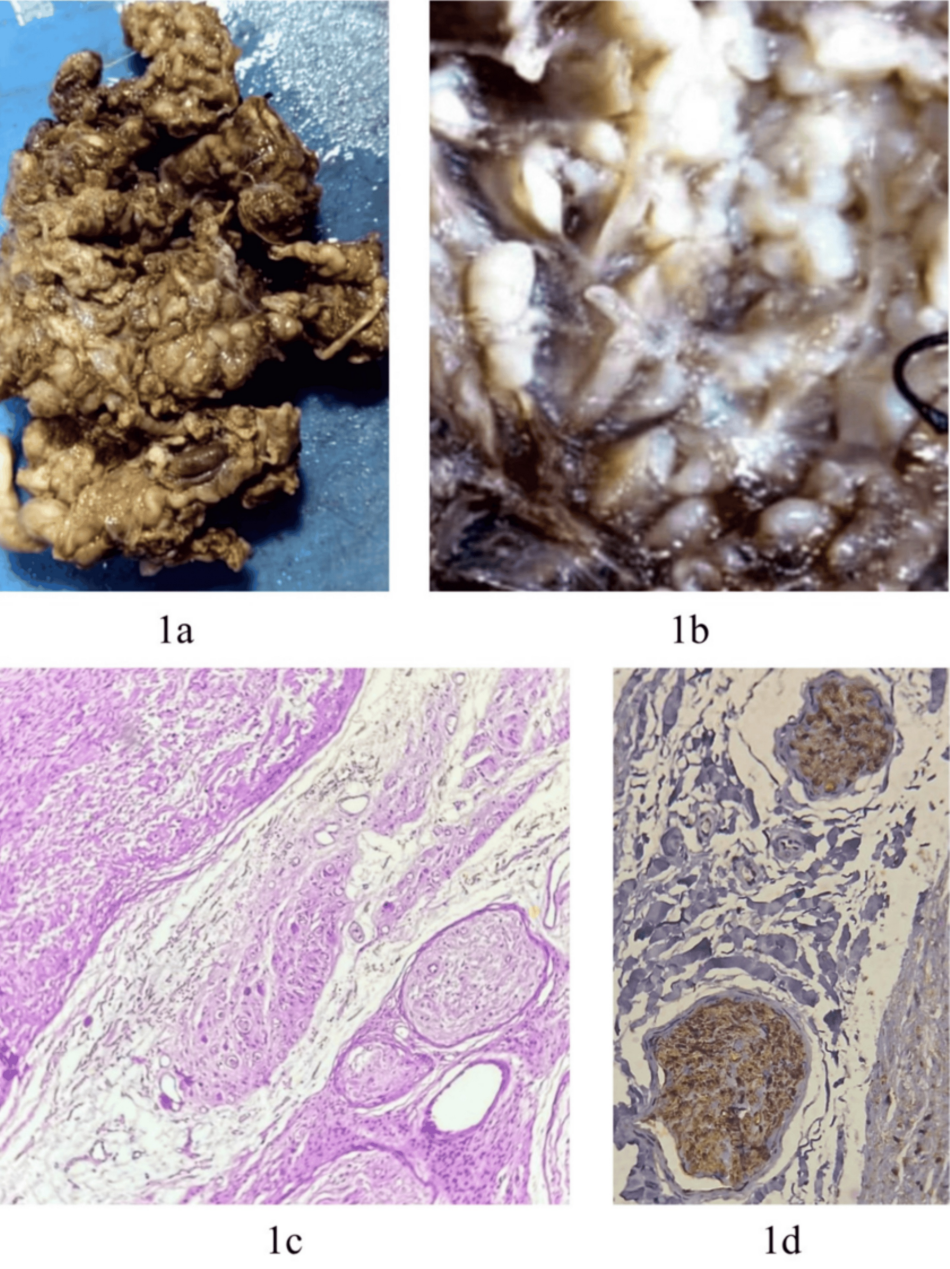
Gross, microscopic, and immunohistochemical findings (a) Gross external examination of the excised solid-cystic mass. (b) Gross cut surface showing a characteristic “bag of worms” appearance. (c) Histopathological examination demonstrating multiple tortuous, proliferating nerve bundles embedded in a myxoid stroma, consistent with a plexiform neurofibroma (hematoxylin and eosin stain, ×20). (d) Immunohistochemical staining showing strong, diffuse S100 protein immunoreactivity in the nerve fibers, supporting the diagnosis of a plexiform neurofibroma (immunohistochemistry, ×20).

The patient underwent surgical excision of the mass. Gross examination revealed a solid-cystic multinodular soft tissue specimen with focal myxoid areas (Figure 1a). On cut section, multiple nerve bundles resembling a “bag of worms” were identified (Figure 1b). Microscopic examination showed a tortuous mass of expanded nerve branches cut in various planes of section. The lesion demonstrated a plexiform growth pattern involving multiple nerve fascicles. No significant atypia, necrosis, or increased mitotic activity was identified. Histopathological findings were consistent with a plexiform neurofibroma (Figure 1c). Immunohistochemistry for S100 protein was positive, confirming the neural origin of the tumor (Figure 1d).

**Figure 2:**
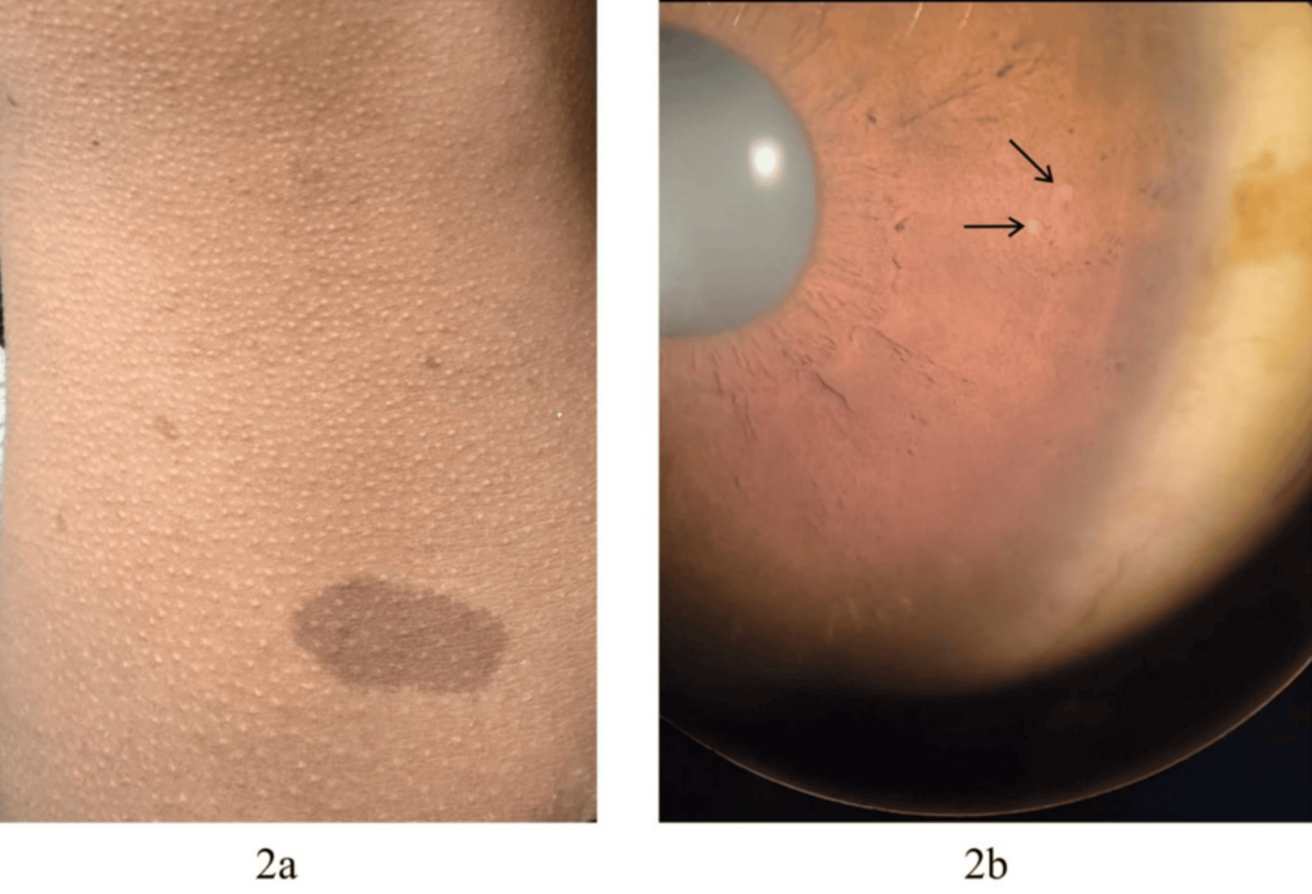
Dermatological and ophthalmic findings (a) Clinical photograph showing several café-au-lait macules and freckling on the patient’s neck. (b) Slit-lamp examination demonstrating multiple iris hamartomas consistent with Lisch nodules.

At follow-up approximately one year later, the patient developed multiple hyperpigmented macules of varying sizes over the body, morphologically consistent with café-au-lait macules (Figure 2a). Ophthalmological examination revealed multiple dome-shaped iris hamartomas consistent with Lisch nodules (Figure 2b). In view of the histologically confirmed plexiform neurofibroma and the subsequent ophthalmological and pigmentary manifestations, a diagnosis of evolving sporadic NF1 was considered.

## Discussion

NF1 is caused by deletions, insertions, nonsense mutations, amino acid substitutions, and splice-site mutations in the NF1 gene, a tumor suppressor gene located in the pericentromeric region of chromosome 17. The NF1 gene encodes an approximately 2,800-amino-acid protein known as neurofibromin. A small portion of neurofibromin possesses sequence homology to RAS GTPase-activating protein, which inactivates RAS. Loss of NF1 gene expression therefore results in increased RAS activity, cell proliferation, and tumorigenesis [1].

Plexiform neurofibroma is a benign but locally infiltrative peripheral nerve sheath tumor that is strongly associated with NF1 and is considered virtually pathognomonic of the disease [1]. These tumors commonly involve cranial, cervical, and major peripheral nerves and may lead to cosmetic deformity, compressive symptoms, and functional impairment [6]. Although most cases occur in association with NF1, isolated plexiform neurofibromas without other stigmata of NF1 have rarely been described [5].

Similar diagnostic challenges have been highlighted by Rajpurohit et al., who reported a cervical plexiform neurofibroma that was clinically diagnosed preoperatively as a hemangioma [5]. Likewise, Tchernev et al. emphasized that plexiform neurofibromas may clinically resemble hemangiomas or lymphangiomas because of their soft, diffuse consistency and infiltrative growth pattern [6].

The differential diagnosis of cervical soft tissue masses in children and young adults includes lymphangioma, hemangioma, schwannoma, lipoma, and other benign or malignant soft tissue tumors. Radiological findings may not always be specific, particularly in diffuse lesions. Histopathological examination, therefore, remains essential for a definitive diagnosis. Typical histological features include tortuous enlarged nerve fascicles embedded in a myxoid stroma with spindle-shaped Schwann cells having wavy nuclei. Immunohistochemically, diffuse S100 positivity supports neural differentiation [1].

The present case is unusual because the patient initially presented only with a cervical plexiform neurofibroma without definitive clinical evidence of NF1 or a significant family history. The delayed appearance of café-au-lait macules and Lisch nodules during follow-up subsequently fulfilled the diagnostic criteria for NF1. This delayed presentation is consistent with the age-dependent penetrance of NF1, in which many diagnostic features evolve progressively during childhood rather than being present at birth or at the initial evaluation [2]. Consequently, young children with an isolated plexiform neurofibroma may not initially satisfy the established diagnostic criteria despite harboring the underlying disorder. Recognizing this evolving clinical pattern is important because failure to consider NF1 at the initial presentation may delay diagnosis, appropriate surveillance for disease-associated complications, genetic counseling, and timely multidisciplinary management.

Plexiform neurofibromas are clinically significant because of their infiltrative nature, tendency for recurrence after incomplete excision, and risk of transformation into a malignant peripheral nerve sheath tumor [1]. Surgical excision remains the primary treatment modality; however, complete removal may be difficult because of diffuse nerve involvement and proximity to vital neurovascular structures [6]. Long-term clinical follow-up is therefore necessary to monitor disease progression, recurrence, and malignant transformation in patients with NF1. This case emphasizes the need for careful longitudinal follow-up of children presenting with an isolated plexiform neurofibroma, even in the absence of other clinical features or a positive family history.

## Conclusions

This case highlights the importance of considering plexiform neurofibroma in the differential diagnosis of cervical soft tissue masses, even in the absence of classic features of NF1 at presentation. Although conclusions from a single case report should be interpreted with caution, this case illustrates that an isolated plexiform neurofibroma may represent the earliest manifestation of evolving NF1 and may clinically mimic other cervical lesions, such as lymphangioma. Clinicians should therefore maintain a high index of suspicion for evolving NF1 in children presenting with an isolated plexiform neurofibroma, even when the family history is negative and other diagnostic features are absent, as additional manifestations may emerge only later in the disease course. Careful long-term follow-up, including dermatological and ophthalmological examinations, is therefore essential for early recognition of evolving NF1 manifestations, timely genetic counseling, and appropriate surveillance for disease-associated complications.
